# Looking for Pathogens in Dust from North Africa Arriving in the French West Indies Using Metabarcoding and Cultivable Analysis

**DOI:** 10.3390/microorganisms12102111

**Published:** 2024-10-21

**Authors:** Yann Reynaud, Andric Gelasse, Luc Multigner, Philippe Quénel, Antoine Talarmin, Stéphanie Guyomard-Rabenirina

**Affiliations:** 1 Unité Transmission Réservoir et Diversité des Pathogènes, Institut Pasteur de Guadeloupe, Guadeloupe, 97139 Les Abymes, France; 2 Institut de Recherche en Santé, Environnement et Travail, UMR_S 1085, INSERM, EHESP, University Rennes, 35000 Rennes, Francephilippe.quenel@ehesp.fr (P.Q.)

**Keywords:** dust, French West Indies, metabarcoding, microbial pathogens

## Abstract

Periodically, the French West Indies receive dust originating from North Africa (NA). Microorganisms associated with desert dust can be transported over long distances through the atmosphere and could represent a means for the remote colonization of new habitats by putatively pathogenic microorganisms. The aim of this study was to determine the diversity and frequency of microbial agents (bacteria, eukaryotes) in NA dusts and the potential threat toward human and/or animal health by comparing microbial air composition during dust events and in control samples. In 2017 and 2018, 16 samples were collected during seven NA dust episodes and there were 9 controls. The microbial composition of the samples was characterized using a cultivable approach and by metabarcoding analyses (16S and 18S). A greater bacterial load and greater diversity were observed during the dust events, and some genera were significantly associated with the events. Some, such as *Geodermatophilus*, can be considered signature species of NA dust. No pathogenic species were found with the cultivable approach, whereas the metabarcoding analyses highlighted the presence of several potentially pathogenic species or known human pathogens such as *Naegleria fowleri*.

## 1. Introduction

Unlike in megacities, where anthropogenic pollution is responsible for high concentrations of particulate matter measuring > 10 µm (PM10), in the Caribbean Basin, high PM10 concentrations are frequently due to large amounts of mineral dust transported each year from North Africa (NA) [1,2]. The archipelago of Guadeloupe (French West Indies) is periodically exposed to desert dust [2], usually between April and October, when the PM10 concentrations are 1.5 times higher than usual, at a mean of 32.9 ± 18.5 μg/m^3^ [2].

Saharan dusts can transport viable microbes and thus be responsible for their dissemination [3]. These microorganisms may survive in their new environment and provoke severe plant diseases, such as the outbreak of the fungus *Aspergillus sydowii*, which caused the death of Caribbean coral reefs in the 1980s [4]. Exposure to desert dust has also been identified as the source of numerous animal diseases, such as aspergillosis in desert locusts [5], and also of human diseases, such as coccidioidomycosis [6], Persian Gulf syndrome [7] and, more recently, COVID-19 [8].

Many studies have demonstrated the association of aerosols with infectious and respiratory diseases. Pathogen-associated molecular patterns, such as those of endotoxins and certain components of the walls of Gram-negative bacteria present in the air, act on the immune system and increase the risk of severe asthma [9]. Areas highly impacted by dust events (DEs), such as the Aral Sea and the Caribbean, have some of the highest recorded incidence rates of asthma worldwide [10]. A study in Guadeloupe showed a significant association between the PM10 concentration (as a proxy for dust events) and the number of emergency consultations for asthma in the Pointe-à-Pitre University Hospital [11]. Several studies have also demonstrated that Saharan dusts exacerbate the severity of COVID-19 [12,13].

Research on Barbados, conducted by the University of Miami (USA) since the late 1960s, has shown an increase in dust flow over the past 25 years, which corresponds to the onset of the current drought in North Africa [14,15,16]. Dust events have been more severe during years of severe drought in the Sahel [17], and droughts are becoming more and more intense in that area, presaging the intensification of dust events in the Caribbean Basin and, putatively, an increased risk of the introduction of pathogens.

Most studies on the microbial composition of North African dusts have been conducted in the Mediterranean region with cultivable and/or non-cultivable (metagenomic, high-throughput sequencing) approaches and have focused mainly on bacterial communities. Furthermore, the number of dust events analyzed was generally low (<5) [18,19,20,21]. In a recent study in the Caribbean, a metagenomic approach was used to characterize five dust events occurring in 2002 and 2008. The microbial composition in these events appeared to be similar from one year to the next, and fungi predominated [22]. Nevertheless, no controls were sampled during non-dust periods, which makes it difficult to discriminate between local airborne microbial communities and those carried by NA dusts. We therefore conducted a study to better understand the diversity and frequency of microbial agents transported by NA dust (eukaryotes, bacteria) and the potential consequences for human and/or animal health by comparing the microbial composition of air during and outside dust events.

## 2. Materials and Methods

### 2.1. Sample Collection

**Figure 1:**
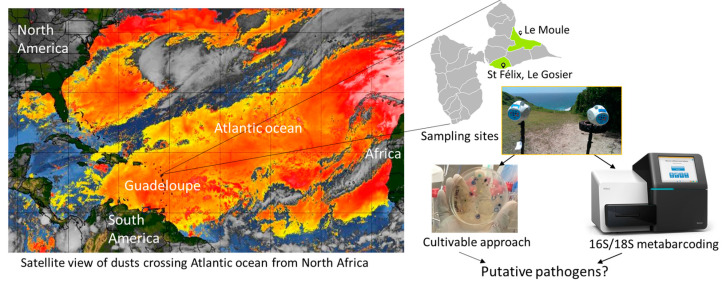
Graphical abstract of the study. Desert dust from North Africa regularly crosses the Atlantic Ocean to settle on the West Indies; increasing particle concentration of dust event is indicated on the map from yellow to orange (Event on 18 July 2018, source: https://www.aoml.noaa.gov/our-research/hurricane-research-division/saharan-air-layer/ (accessed on 10 March 2020)). These dusts are able to transport viable microbes that can have an effect on human and animal health but also on the environment. We undertook this work to characterize these microbes and search for pathogens. After dust sampling on Petri dishes and filters, microorganisms were identified by two complementary approaches, a cultivable method and by 16S and 18S rDNA sequencing. No pathogenic microorganisms were found with the cultivable method, but we could evidence the presence of the genetic material of several putative pathogenic species.

Between March and November 2017 and 2018, 16 dust samples (DSs) were collected during seven NA dust episodes and 9 control samples were collected (CSs, i.e., non-dust events). Sampling sites were chosen according to the wind direction and in order to limit introduction of microorganisms from the terrestrial pollution. Samples were taken on the cliffs of Saint Félix, Gosier (16.196707, −61.464075, elevation 7 m) to represent south-easterly trade winds (7 DSs and 5 CSs), and on the heights of Le Moule (16.3569, −61.39213, elevation 62 m) for north-easterly trade winds (7 DSs and 4 CSs) (Figure 1).

The days of sampling were chosen according to air quality data forecasts provided by the association for air quality in Guadeloupe (Gwadair). An index, previously described (Quénel et al., 2021), was derived empirically from the 24 h mean values of PM10 to define days without and with NA dust events: I0 (no NADE) if the 24 h mean value PM10 was ≤27 µg/m^3^, I1 (light NADE) for 27 < PM10 ≤ 38 µg/m^3^, I2 (moderate NADE) for 38 < PM10 ≤ 54 µg/m^3^ and I3 (intense NADE) for PM10 > 54 µg/m^3^. Nine DSs were collected during intense NADE, four during moderate NADE and one during light NADE. The mean value of PM10 was 68.46 µg/m^3^ (min 30.3, max 106.7) during NADE and 17.5 µg/m^3^ (min 6.7, max 27) outside NADE.

Airborne dust was sampled with Microflow air bio-collectors (Aquaria, Kuala Lumpur, Malaysia), which have 380 1 mm pores. For cultivable bacteria, the agar media were placed directly in the collector. Three media were used, a non-selective nutrient agar (trypticase soy agar (TSA) (Biokar Diagnostic, Pantin, France), a plate count agar (PCA) (Biokar Diagnostic, Pantin, France) for enumeration of aerobic bacteria, and a non-selective chromogenic medium (Chromagar Orientation (CO), CHROMagar, Paris, France)). TSA and PCA plates were placed in the collector for 30 s at a flow rate of 30 L/min, and CO plates were placed for 7.5 min at a flow rate of 120 L/min. The media were incubated for 24–48 h at 36 ± 2 °C.

For non-cultivable microorganisms, a sterile filter of 0.2 µm pore size was deposited on the surface of an agar plate. The plate was then placed in the collector for 4 h at a flow rate of 120 L/min. At the end of sampling, the filters were placed in 45 mL of phosphate-buffered saline (PBS) buffer in a 50 mL Falcon vial and conserved at −80 °C until DNA extraction.

### 2.2. Numeration and Identification of Cultivable Bacteria

After 24–48 h of incubation, the number of colony-forming units (CFU) was counted on each medium, and the number of CFU/m^3^ was calculated by reducing the number of colonies counted on each medium to the volume of air sampled. Each colony was isolated and identified by matrix-assisted laser desorption/ionization time-of-flight mass spectrometry on an Axima Performance (Shimadzu Corp., Osaka, Japan).

### 2.3. Statistical Analyses for Cultivable Microorganisms

We used descriptive statistics to compare the proportions of the CFU values in samples. The average CFU/m^3^ values obtained for DSs and CSs on each medium were compared using the Wilcoxon nonparametric test for paired series.

### 2.4. Detection of Total Microorganisms

The filters were thawed under a level-2 biological safety cabinet in a Petri dish containing PBS buffer. The mesh at the rear of the filter was removed, and the top of the filter was cut into small pieces with a sterile scalpel. The remainder was centrifuged at 8000 rpm for 10 min at 4 °C in order to concentrate the bacteria in suspension. After the supernatant was discarded, the filter pieces and the buffer were centrifuged at 8000 rpm for 10 min at 4 °C. Only the pellet was retained after centrifugation.

Total DNA was extracted from the pellet with a NucleoSpin^®^ Soil Kit (Macherey-Nagel, Hoerdt, France) according to the manufacturer’s instructions.

The quality of the extracted DNA was controlled in a Jenway Genova Nano spectrophotometer (London, UK).

DNA extracts were stored at −20 °C until they were sent to the biomics platform of the Institut Pasteur in Paris for the next sequencing steps.

### 2.5. 16 S rDNA and 18S rDNA Metabarcoding Analyses

A total of 23 samples were included for 16S rDNA metabarcoding (n = 14 samples from dust events, DSs, n = 9 non-dust events, i.e., control samples, CSs) and 25 samples for 18S rDNA metabarcoding (n = 16 DSs, n = 9 CSs).

Amplicon libraries targeting the V3-V4 variable regions of the 16S rRNA gene and the V4 region of the 18S rRNA gene were prepared in a Nextera XT kit (Illumina, San Diego, CA, USA) and sequenced on a MiSeq system (Illumina) using the Biomics platform (Institut Pasteur, Paris, France). Paired-end reads (300 bp) were trimmed (library adapter and primer sequence removal) and filtered with AlienTrimmer software v0.4.0 [23] at a Phred quality score threshold of 28 on a minimum length of 70 nucleotides, resulting in a total of 5 202 074 reads for 16S (mean, 22 6177; range, 29 038–882 358) and 4 077 454 reads for 18S (mean, 163 098; range 30 144–322 994). Denoising, merging and chimera detection were performed with the DADA2 software package [24] implemented in the QIIME 2 pipeline (version 2020.2) [25] via q2-dada2 plugin. As merging for 18S reads failed, only forward reads were used for the next analyses. DADA2 allows for the identification of fine-scale variation through the characterization of amplicon sequence variants (ASVs). A total of 1237 ASVs and 697 ASVs were characterized for the 16S and 18S analyses, respectively. The whole process retained 2 792 393 reads (mean, 121 408; range, 13 353–49 8136; mean length, 410 bp) for 16S and 2 917 398 reads (mean, 116 695; range 24 219–243 630; length, 240 bp) for 18S.

Taxonomy was assigned to ASVs with the q2-feature-classifier (a classify-sklearn naïve Bayes taxonomy classifier based on machine learning) against the Silva 132 99% database [26]. The ASV table was normalized by the DESeq2 method [27], which was modified in the SHAMAN pipeline [28] in order to better account for matrix sparsity, as suggested by McMurdie and Holmes [29]; the null normalization method was used, in which cells with null values were excluded from computation of the geometric mean. Rarefaction curves were computed to evaluate the quality of the deep sequencing effort. Alpha diversity was calculated with the Shannon, Simpson, inverse Simpson and alpha indexes. Principal coordinate analysis (PCoA) was performed, based on the Bray–Curtis distance at the taxonomic genus level, to evaluate between-sample dissimilarities for the tested variables: the date of whole-genome sequencing (WGS) (i.e., February 2018 vs. November 2018) and sampling conditions (i.e., DS dust sample vs. CS control/regular sample). The effects of these variables on beta diversity were tested with permutational multivariate ANOVA methods (PERMANOVA) with 999 permutations of the Bray–Curtis distance matrix.

The generalized linear model was used to detect differences in abundance between dust samples and regular conditions at the genus level. These analyses were performed separately for samples sequenced in February and in November 2018. Corresponding *p* values were adjusted for multiple testing according to the Benjamini–Hochberg correction.

For identification of species significantly associated with dust events (and with a low level of species assignation in the global matrix: 27% for 16S and 11% for 18S), our approach was to apply the same statistical tests to individual ASVs only when a genus was assigned. In a second step, ASVs significantly associated with dust events were taxonomically assigned to the National Center for Biotechnology Information (NCBI) database with the BLASTN algorithm [30].

## 3. Results

### 3.1. Cultivable Bacteria

**Figure 2:**
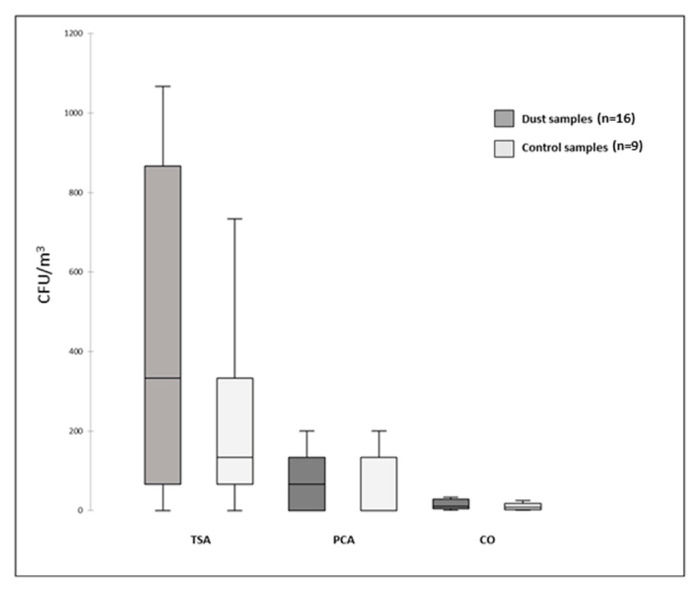
Number of CFU/m^3^ of bacteria in air samples on different media (TSA, trypticase soy agar; PCA, plate count agar; CO, chromagar orientation).

The highest bacterial loads were observed on TSA media, whereas the bacterial loads obtained on CO media were relatively low. Overall, we observed a lower growth on PCA than on TSA medium, which is more nutrient-enriched; no significant difference was found with the control samples (Figure 2).

**Figure 3:**
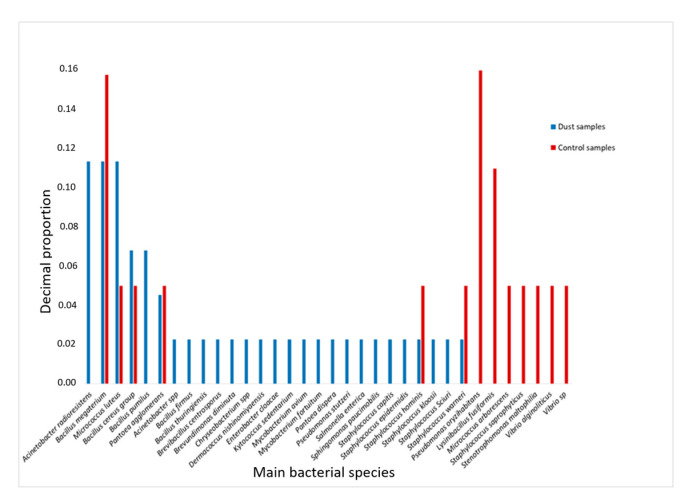
Frequency of the main bacterial species isolated according to sampling condition (during dust events and in control samples).

During dust events, 106 bacterial strains were isolated, including 44 (41.5%) that could be identified by mass spectrometry, of which 27 (61.3%) were Gram-positive and 15 (34.1%) were Gram-negative. *Bacillus* was the most frequent genus, with 13 strains identified, followed by *Acinetobacter* (n = 6) and *Staphylococcus* (n = 6) (Figure 3). *Acinetobacter* radioresistens, *Bacillus megaterium* and *Micrococcus luteus* were the species most commonly isolated. Six species in the *Staphylococcus* genus and five in the *Bacillus* genus were identified. *A. radioresistens* and *M. luteus* were the species most frequently found in all the samples (n = 5/14 DS). Fewer bacteria were isolated in the control samples (n = 67), and fewer strains could be identified by mass spectrometry (n = 19, 28.3%); of these, 11 were Gram-positive. As observed during the dust events, *Bacillus* and *Staphylococcus* were the most frequent genuses, whereas no strains belonging to *Acinetobacter* were isolated. Bacteria in the genus *Vibrio* were found only in the control samples (Figure 3).

### 3.2. Metabarcoding Analyses

The rarefaction curves (Figure S1) reached a plateau, indicating that bacterial diversity was satisfactorily detected. An analysis of the beta diversity calculated according to the Bray–Curtis dissimilarity revealed a clear division of the data according to the date of the WGS in both the 16S and 18S analyses (February 2018 vs. November 2018, PERMANOVA *p* value < 0.001) (Figure S2); therefore, further analyses were performed to determine the genera and species associated with the Saharan dust on two separate datasets corresponding to the dates of the WGS.

#### 3.2.1. Bacterial Communities Associated with NA Dusts in Guadeloupe

Of the 1237 ASVs, 97.74% were assigned at the family level, 74.29% at the genus level and 27.81% only at the species level. A total of 305 genera were identified (corresponding to 919 ASVs).

**Figure 4:**
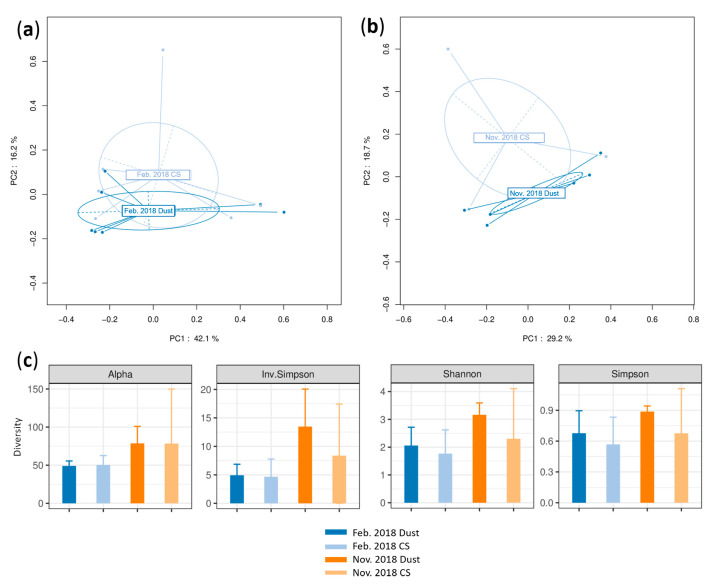
Beta and alpha diversity analyses of bacterial communities at genus taxonomic level. PCoA plots according to sampling condition (during dust events or in control samples, CSs) based on a Bray–Curtis dissimilarity matrix for (**a**) the February 2018 dataset (indicated by Feb. 2018) and (**b**) the November 2018 dataset (indicated by Nov. 2018). PERMANOVA test based on sample type yielded *p* = 0.498 and 0.443, respectively; 58.3% and 47.9% of variations were explained by the first two PC1 and PC2 axes for the February and November datasets, respectively. (**c**) Alpha diversity analysis based on alpha, inverted Simpson, Shannon and Simpson indices during dust events and in control conditions; error bars represent 95% confidence intervals.

No division of data was seen between the DSs and CSs according to the PCoA in the February and November 2018 datasets (PERMANOVA *p* = 0.498 and 0.443, respectively) (Figure 4a,b).

The overlapping confidence intervals in the alpha diversity indicate that the diversity of the DSs and CSs did not differ according to any index (Figure 4c); nevertheless, a tendency to a greater diversity for the DSs was seen, especially in the November 2018 dataset.

**Figure 5:**
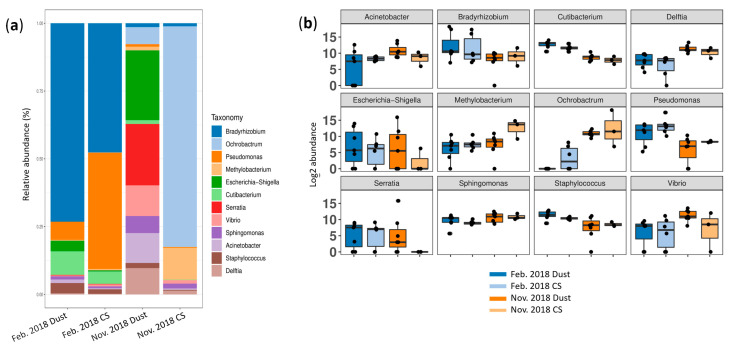
Dominant bacterial genera according to sample type (during dust events or control samples, CSs) for the February 2018 dataset (indicated by Feb. 2018) and November 2018 dataset (indicated by Nov. 2018). (**a**) Bar plot of relative abundance. (**b**) Box plot of log2 abundance.

With regard to the relative abundance of the 12 dominant genera, the DSs and CSs for February 2018 were similar, with a predominance of the same genera: *Bradyrhizobium* (73.2% and 47.6%, respectively), *Pseudomonas* (6.7% and 42.9%), *Cutibacterium* (8.7% and 4.6%), *Escherichia*/*Shigella* (3.9% and 0.4%) and *Staphylococcus* (3.8% and 1.7%). In the November 2018 data, more varied bacterial communities were observed, the predominant genera being *Ochrobactrum* (81.4%) and *Methylobacterium* (11.7%) in the CSs and a more diverse community in the DSs with a predominance of *Escherichia*/*Shigella* (25.7%), *Serratia* (22.6%), *Vibrio* (11.3%), *Acinetobacter* (11%), *Delftia* (9.7%), *Ochrobactrum* (6.3%) and *Shingomonas* (6.3%) (Figure 5).

Although the community structures were similar in the DSs and CSs according to the PCoA results, we tested whether the abundance of certain bacterial taxa differed. The relative abundance of the 305 bacterial genera differed statistically significantly for 11, which were associated with dust events: *Neptunomonas*, *Donghicola* and *Solibacillus* in the February 2018 dataset and *Serratia*, *Geodermatophilus*, *Hyphomicrobium*, *Hydrocarboniphaga*, *Fusobacterium*, *Paraclostridium*, *Methylibium* and *Enterococcus* in the November 2018 dataset (log2 fold change range, 6.55–8.85; *p* < 0.1) (Table S2). At the species level, 160 ASVs were significantly associated with the NA dust events (101 in the February 2018 dataset and 59 in the November 2018 dataset) (Table S2). Species in the *Acinetobacter* genus were among those significantly associated with dust, which is consistent with what was observed with the cultivable approach. Other species such as *Brevundimonas diminuta* and *Staphylococcus epidermidis* were also isolated exclusively from the dust samples. Of the ASVs, 52% were found in only one sample and should therefore be confirmed. The corresponding ASV sequences were further assigned against the NCBI database by BLASTN, which allowed for the identification of 35 bacterial species; 54 ASVs were assigned to two or more species, and the remaining ASVs were blasted with uncultured bacterial species. Globally, the blast results indicate a mean nucleotide identity of 99.67% (on a mean query length of 415 bp).

#### 3.2.2. Eukaryotic Communities Associated with NA Dusts in Guadeloupe

**Figure 6:**
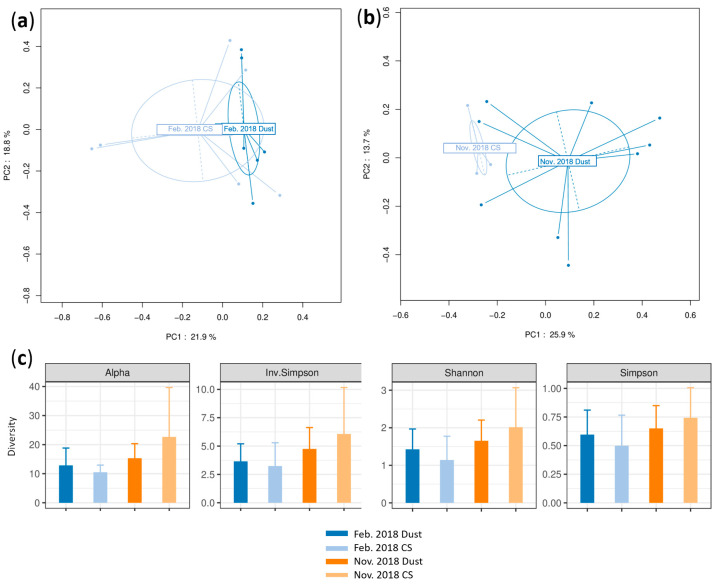
Beta and alpha diversity analyses for eukaryotic communities at genus taxonomic level. PCoA plots according to sampling condition (during dust events or in control conditions, CSs) based on a Bray–Curtis dissimilarity matrix for (**a**) the February 2018 dataset (indicated by Feb. 2018) and (**b**) the November 2018 dataset (indicated by Nov. 2018); a PERMANOVA test based on sample type yielded *p* = 0.603 and 0.156, respectively; 40.7% and 39.6% of variations were explained by the first two PC1 and PC2 axes for the February 2018 and November 2018 datasets, respectively. (**c**) Alpha diversity analysis based on alpha, inverted Simpson, Shannon and Simpson indexes during dust events and in control regular conditions; error bars represent 95% confidence intervals.

In the 18S rDNA analyses, only 32.86% of the 697 ASVs studied were assigned at the family level, 22.67% at the genus level and 11.05% at the species level. A total of 96 genera were identified. As in the study of the bacteria, no difference was found in the PCoA between the DSs and CSs taken in February 2018 and in November 2018 (PERMANOVA *p* = 0.603 and 0.156, respectively) (Figure 6a,b). The alpha diversity also did not differ between the DSs and CSs in any index (Figure 6c); nevertheless, there was a tendency to greater diversity in the dust samples in the February 2018 dataset.

**Figure 7:**
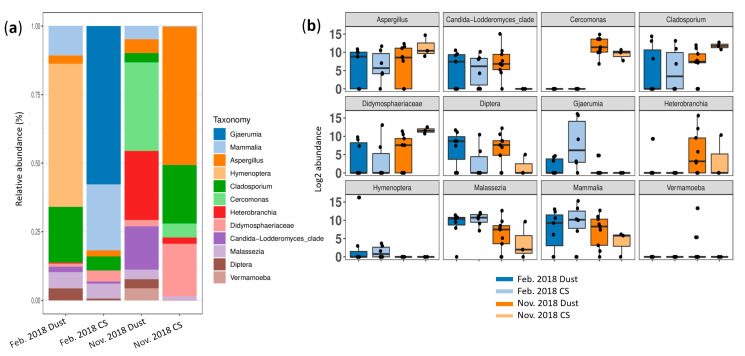
Dominant eukaryotic genera according to sample type (during dust events or control samples, CSs) for the February 2018 dataset (indicated by Feb. 2018) and November 2018 dataset (indicated by Nov. 2018). (**a**) Bar plot of relative abundance. (**b**) Box plot of log2 abundance.

With regard to the relative abundance of the 12 dominant genera in the February 2018 dataset, we observed a predominance in the DSs of *Hymenoptera* (52.2%) and *Cladosporium* (20.3%), while *Gjaerumia* (57.7%) and *Mammalia* (24.1%) were more abundant in the CSs. In the November 2018 data, the DSs contained mainly *Cercomonas* (32.3%), *Heterobranchia* (25.2%) and *Candida* (15.8%), while *Aspergillus* (50.5%), *Cladiosporium* (21.3%) and *Didymosphaeriaceae* (19.1%) predominated in the CSs (Figure 7).

We also determined whether specific genera were differentially abundant. The overall comparison of the relative abundance of the 96 eukaryotic genera revealed statistically significant differences for 14/96 taxa associated with the dust events: *Hymenoptera*, *Cyclidium*, *Aplanochytrium*, *Panus*, *Graphium*, *Jaminaea*, *Arcocellulus* and *Physalacria* in the February 2018 dataset and *Candida-Lodderomyces*, *Vermamoeba*, Naegleria, *Terebellida*, *Hemimycena* and *Olpidium* in the November 2018 samples (log2 fold change range 6.53–8.94, *p* < 0.1) (Table S4). At the species level, the NA dust was significantly associated with 29 ASVs (17 for the February 2018 dataset and 12 for the November 2018 dataset) (Table S4). Of these ASVs, 69% were observed in only one sample and should therefore be confirmed. The corresponding ASV sequences assigned against the NCBI database with BLASTN allowed for the clear identification of 13 species, while 14 other ASVs were assigned to 2 or more species (mean nucleotidic identity, 99.23%).

## 4. Discussion

We observed a high inter-sample variation in the bacterial counts, in particular on the TSA and PCA media with a cultivable approach, which can be explained by our sampling method, as the Petri dishes were placed for only a short time in the air sampler in order to prevent medium invasion, and the air drawn in was probably more or less loaded with particles according to the wind force and direction. This high variation precluded any significant differences between the bacterial loads in the DSs and CSs. Nevertheless, the mean bacterial loads were higher during the dust events except for in the PCA, in which one CS had a particularly high bacterial load.

We used mass spectrometry to identify cultivable bacteria. High proportions of bacteria (58.5% in DSs and 71.7% in CSs) could not be identified, revealing the limitations of this technique. New isolates can be identified by MALDI-TOF mass spectrometry only if the spectral database contains peptide mass fingerprints of the type strains of specific genera, species, subspecies and strains [31]. Studies in which the amplification of bacterial 16S rRNA was used obtained the better identification of cultivable strains [32,33], whereas strains could not be identified in a study conducted in Senegal with conventional biochemical tests [34].

Under all the conditions, the genus most frequently isolated in our samples was *Bacillus*. Bacteria of this genus were also the most frequently isolated from dust samples collected in the Caribbean [17] and samples harvested from the Bodélé Depression, off the coast of Cape Verde, in sand from Chad [32] and on Mediterranean coasts [35]. Bacteria of the genus *Bacillus* are widely found in soil, where they can form dormant spores under adverse environmental conditions (heat, chemicals or sunlight), which can invade dust particles, thus allowing their dispersion into new environments, in which they can proliferate again if they find favorable conditions. One of the most frequent species of *Bacillus* was *B. megaterium*. These bacteria are involved in the mineralization of organic phosphate compounds present in soil to make phosphorus available to plants [36].

The predominance of Gram-positive bacteria is probably due to their high resistance to desiccation and sunlight. *Acinetobacter radioresistens*, a Gram-negative species, was also among the most frequently isolated species during dust events. This species has remarkable tolerance to radiation and desiccation and also to hydrogen peroxide and ultraviolet irradiation [37], allowing it to survive in hospitals [38]. *A. radioresistens* has also been identified as the progenitor of *bla*_OXA23_-like genes, which are presumably transposed in a plasmid and transferred to *A. baumanii*. *bla*_OXA23_ genes are a major source of carbapenem resistance in *A. baumanii* worldwide [39].

The second approach we used to explore microbial communities associated with NA dusts in Guadeloupe was 16S and 18S metataxonomic analyses. First, a clear difference in microbial structure according to the date of the WGS (February 2018 vs. November 2018) was observed with the PCoA (Figure S2). The reason for this is unclear but might be linked to technical factors such as the sequencing run and DNA extraction batch or to differences in bacterial and eukaryotic communities according to the year or season. Waters et al. also reported the absence of clustering between Saharan dust metagenomes sampled at different times in the Caribbean [22]. They hypothesized that the intensity of the DE and the long transit across the Atlantic Ocean affect the sample composition differently. We analyzed two separate datasets corresponding to the date of the WGS.

No clear differences in the abundance of genera were observed between the DSs and CSs in the February 2018 dataset while in those from November 2018, *Escherichia/Shigella* (25.7%), *Serratia* (22.6%), *Vibrio* (11.3%), *Acinetobacter* (11%), *Delftia* (9.7%) and *Shingomonas* (6.3%), were more abundant during the dust events (Figure 5). Of these, only *Serratia* was statistically associated with the dust (log2-fold change, 8.853; *p* adjusted = 0.014) (Table S3). *Serratia* was also identified among viable bacteria in samples collected during DEs in Dakar, Senegal [34]. Among the 11 other genera identified in our study, only *Geodermatophilus*, which showed a log2-fold change of 6.554 (*p* adjusted = 0.089), was the only one previously recovered from Saharan dust samples, in the Dolomite Alps [20] and in the Eastern Mediterranean [19]. This genus was also identified as one of the most abundant in sand collected in Chad [32]. Most species of *Geodermatophilus* have been isolated from desert soils [40].

In order to determine whether putatively pathogenic bacterial species are significantly associated with NA dust, we tested ASVs corresponding to their assigned genus and found that 160 were potentially linked to dust; these were further blasted against the NCBI database (mean nucleotidic identity, 99; 67 bp) (Table S4). Of these ASVs, 52% were recovered in only one dust sample, and the results should be confirmed. Few ASVs were assigned to a single potentially pathogenic species. With regard to human pathogens, we identified *Aureimonas altamirensis*, a rare pathogen associated with bloodstream infections and pleural effusion [41,42], which was previously isolated in a desert environment (the Gibson desert in Australia) [43]. The opportunistic pathogens found that were previously detected were *Bacillus circulans* [44], *Rothia mucilaginosa* [45] and *Moraxella atlantae* [46]. Species responsible for nosocomial infections, such as *Staphylococcus epidermidis* [47] and *Streptococcus salivarius*, were also found [48]. 

Of the animal pathogens found, *Acinetobacter johnsonii* has been described as an emerging fish pathogen [49], and *Lysinibacillus sphaericus* is an entomopathogenic bacteria [50].

Of the ASVs assigned to two or more species, putatively pathogenic bacteria also matched the NCBI database, such as *Acinetobacter lwoffii*, *Bacillus thuringiensis*, *E. coli*, *Shigella flexneri* and *Staphylococcus aureus*; however, the ASVs could not be assigned clearly to a single species for the corresponding genus because the fragments of 16S rDNA (mean size, 410 bp) were too small, and no conclusion could be drawn about the other pathogenic bacteria associated with the Saharan dusts.

With regard to eukaryotes associated with NA dusts, we observed different communities during the dust events and in the control samples, and in the February 2018 and November 2018 datasets. In February 2018, *Hymenoptera* (52.2%) and *Cladosporium* (20.3%) predominated; *Cladosporium* has already been associated with dust in Saudi Arabia [51]. In November 2018, *Cercomonas* (32.3%), *Heterobranchia* (25.2%) and *Candida* (15.8%) predominated (Figure 7).

At the species level, we characterized a total of 29 ASVs significantly linked to dust events (19 were recovered in one sample) (Table S4). One ASV found in great abundance (51,491 reads) in a single dust sample showed 100% nucleotidic identity with *Candida parapsilosis* and *C. orthopsilosis*; the former is a major cause of disease in immunosuppressed individuals and in premature neonates, while *C. orthopsilosis* is more rarely associated with infection [52].

Another ASV recovered from one sample (2203 reads) was blasted with *Naegleria australiensis* (100% nucleotidic identity), a free-living amoeba which is putatively pathogenic [53]. A second ASV was identified in one dust sample (167 reads) and blasted with two strains of *Naegleria fowleri* from Thailand and China (Genbank accession numbers KT375442 and KY062165, 100% nucleotidic identity, *p* = 0.13); the strain from Thailand also matched *N. fowleri* found in China and belonged to genotype 2 [54], a genotype previously identified in environmental samples in Guadeloupe (unpublished). *N. fowleri* is responsible for rare but often fatal amoebic meningoencephalitis [55] (Grace et al., 2015), such as in a child who swam in geothermal water in Guadeloupe [56]. Our team has shown that *N. fowleri* can be isolated from geothermal and stream waters in most parts of Basse-Terre island in Guadeloupe [57], and a more recent large geographical survey with 18S metabarcoding showed that *N. fowleri* is also present on Grande-Terre, the main island of Guadeloupe, as well as on the Les Saintes islands [58]. *N. fowleri* was also isolated from harmattan winds between Sahara and Nigeria [59]. This free-living amoeba can resist drastic environments by forming dormant resistant cysts. It is possible, therefore, that *N. fowleri* could cross the Atlantic Ocean to Guadeloupe on trade winds and then move through the archipelago until it finds suitable environmental conditions in which to develop and prosper. This possibility will be explored further in our laboratory, where we will quantify *N. fowleri* from dozens of dust samples and attempt the cultivation of this amoeba species directly from NA dusts.

Another free-living amoeba statistically associated with the dusts was *Vermamoeba vermiformis* (formerly *Hartmannella vermiformis*), which is also capable of encystment under harsh conditions. Although this species appears rarely to be pathogenic for humans (causing mainly keratitis), it could constitute a potential hazard, as it can act as a vehicle and reservoir for bacterial pathogens (endocytobionts) [60].

Some other ASVs associated with two or more species could be pathogenic, such as, for example, *Malassezia* [61] and *Cladosporium* [62]. The latter have been isolated from air samples collected in various regions affected by African dust storms (northern Caribbean, Mali, Atlantic Ocean) [63,64] and may inhibit the growth of vegetables such as lettuce and cucumber.

## 5. Conclusions

We identified the potential Saharan origin of NA dusts in Guadeloupe in 2017 and 2018 by characterizing the genera and species common in that region. The great diversity of the microorganisms observed in the DSs also indicates the contribution of new taxa to the microbiome of the natural air of Guadeloupe. No pathogen species were found by the cultivable approach, whereas the metabarcoding analyses revealed the presence of several potentially pathogenic species. Most were opportunistic or rare pathogens and were present in only a few samples. Nevertheless, the putative identification of some species, such as *Naegleria fowleri*, is of main concern. Further studies are needed to confirm and quantify the abundance of live pathogens in NA dust samples reaching Guadeloupe. The first step would be to increase the sampling effort for metabarcoding studies over a greater number of monitoring years, and then to develop metagenomic approaches to explore the diversity of potential pathogens within NA dust with greater resolution.

## Data Availability

Fastq files have been deposited in NCBI-SRA public archives under BioProject accession number PRJNA940153.

## Supplementary Materials

The following supporting information can be downloaded at https://www.mdpi.com/article/10.3390/microorganisms12102111/s1. Figure S1: Rarefaction curves of ASVs for each sample: (A) bacterial rarefaction and (B) eukaryotic rarefaction. Figure S2: PCoA plots of bacterial (A) and eukaryotic (B) between-sample dissimilarities according to date of WGS at genus taxonomic level: PCoA was computed from Bray–Curtis distance, 40.2% and 26.4% of variations were explained by the first two PC1 and PC2 axes for bacterial and eukaryotic PCoA, respectively; PERMANOVA test based on the sample date of WGS yielded *p* = 0.001. Table S1: Date, location and characterization of air quality of sampling. Table S2: Bacterial genera significantly associated with NA dust. Log2-fold change > 1; *p* < 0.1. Table S3: Bacterial species significantly associated with NA dust and ASV BLASTN assignment against NCBI database; *p* < 0.1. Only the first three BLASTN results are presented if several results were possible for the same nucleotidic identity and query coverage. Table S4: Eukaryotic genera significantly associated with NA dust. Log2-fold change > 1; *p* < 0.1. Table S5: Eukaryotic species significantly associated with NA dust and ASV BLASTN assignments against NCBI database; *p* < 0.1. Only the first three BLASTN results are presented if several results were possible for the same nucleotidic identity and query coverage.
