# Supplementary figures and images for: Looking for Pathogens in Dust from North Africa Arriving in the French West Indies Using Metabarcoding and Cultivable Analysis
Source: Microorganisms. 2024 Oct 21;12(10):2111. doi: 10.3390/microorganisms12102111 (PMC11510511; doi:10.3390/microorganisms12102111)

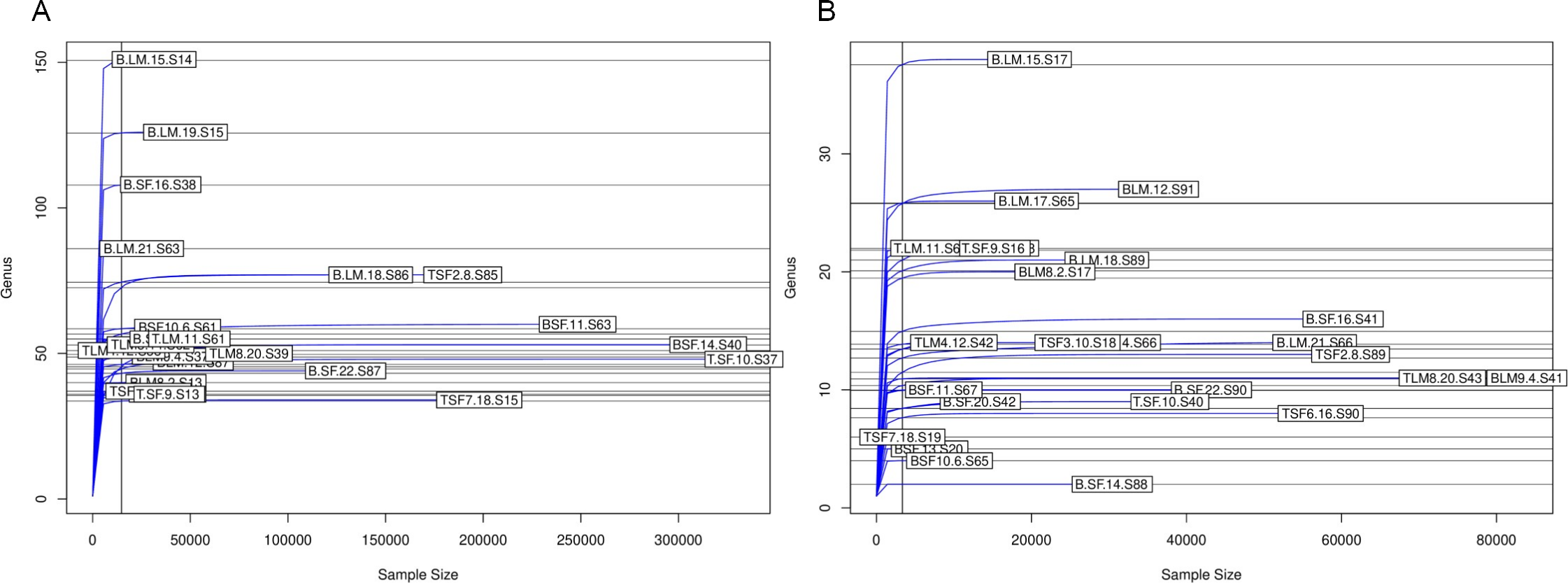

Supplement: Supplementary file 1 [file microorganisms-12-02111-s001.zip › FigureS1.tiff]

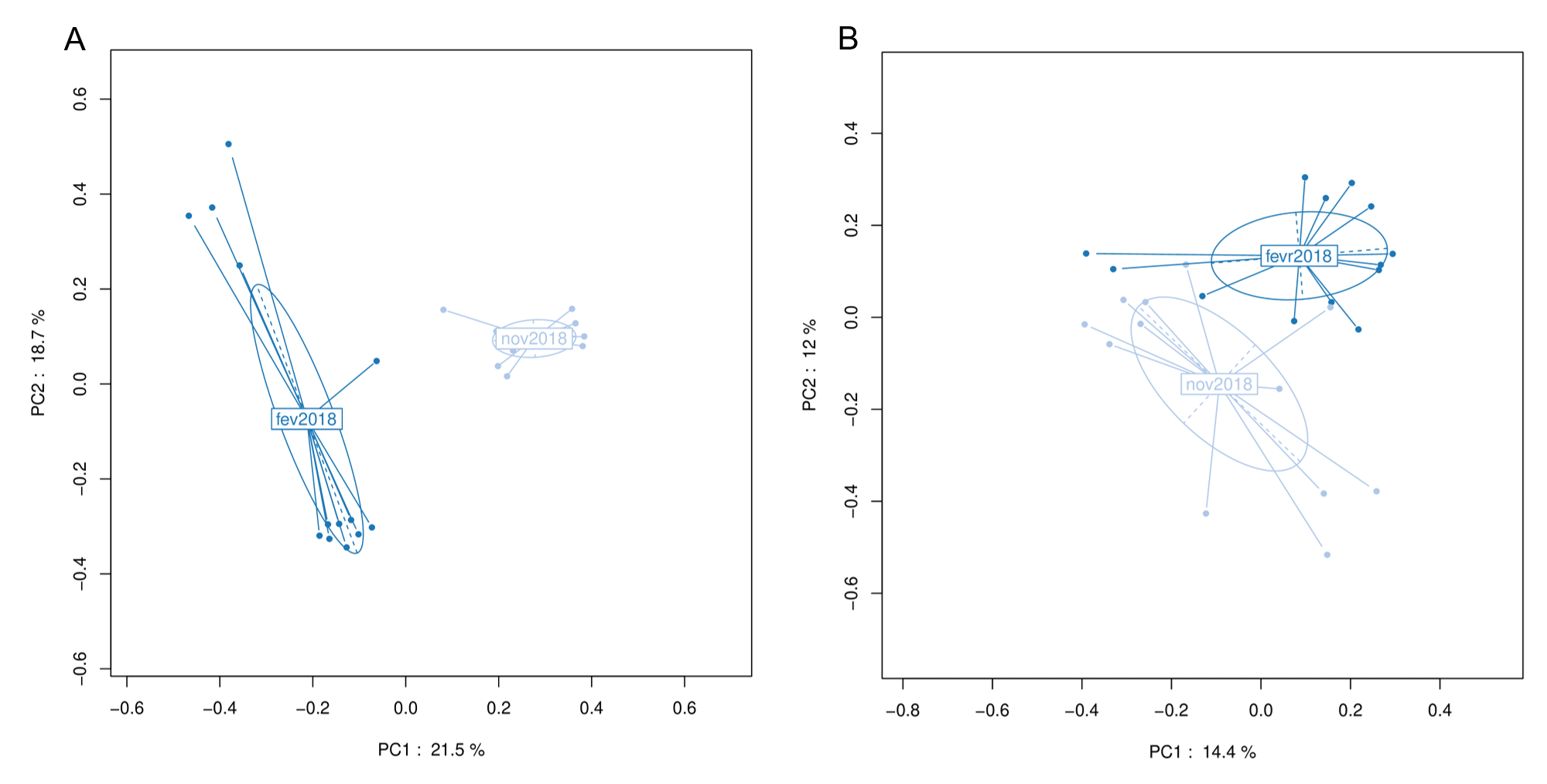

Supplement: Supplementary file 1 [file microorganisms-12-02111-s001.zip › FigureS2.tif]
